# Multiscale in-situ characterization of static recrystallization using dark-field X-ray microscopy and high-resolution X-ray diffraction

**DOI:** 10.1038/s41598-024-56546-9

**Published:** 2024-03-14

**Authors:** Sangwon Lee, Tracy D. Berman, Can Yildirim, Carsten Detlefs, John E. Allison, Ashley Bucsek

**Affiliations:** 1 Department of Mechanical Engineering, University of Michigan, Ann Arbor, MI USA; 2 Department of Materials Science and Engineering, University of Michigan, Ann Arbor, MI USA; 3 European Synchrotron Radiation Facility, Grenoble Cedex 9, France

**Keywords:** Engineering, Materials science

## Abstract

Dark-field X-ray microscopy (DFXM) is a high-resolution, X-ray-based diffraction microstructure imaging technique that uses an objective lens aligned with the diffracted beam to magnify a single Bragg reflection. DFXM can be used to spatially resolve local variations in elastic strain and orientation inside embedded crystals with high spatial (~ 60 nm) and angular (~ 0.001°) resolution. However, as with many high-resolution imaging techniques, there is a trade-off between resolution and field of view, and it is often desirable to enrich DFXM observations by combining it with a larger field-of-view technique. Here, we combine DFXM with high-resolution X-ray diffraction (HR-XRD) applied to an in-situ investigation of static recrystallization in an 80% hot-compressed Mg–3.2Zn–0.1Ca wt.% (ZX30) alloy. Using HR-XRD, we track the relative grain volume of > 8000 sub-surface grains during annealing in situ. Then, at several points during the annealing process, we “zoom in” to individual grains using DFXM. This combination of HR-XRD and DFXM enables multiscale characterization, used here to study why particular grains grow to consume a large volume fraction of the annealed microstructure. This technique pairing is particularly useful for small and/or highly deformed grains that are often difficult to resolve using more standard diffraction microstructure imaging techniques.

## Introduction

With continuing improvements to facilities, detectors, and lenses, synchrotron X-ray techniques are reaching higher and higher spatial, angular, and temporal resolutions. In the case of diffraction microstructure imaging (DMI) techniques, higher spatial resolution results in an ability to measure local, intragranular variations in orientation, elastic strain, and phase. High-resolution DMI techniques include Bragg coherent diffraction imaging (BCDI)^1^, scanning 3D X-ray diffraction (scanning 3DXRD)^2^ or point-focused high-energy diffraction microscopy (pf-HEDM)^3^, and dark-field X-ray microscopy (DFXM). In particular, DFXM is a novel full-field DMI technique that uses a compact refractive lens (CRL) objective to magnify one particular Bragg reflection, resulting in the ability to map internal orientation and elastic strain spread across a crystal or grain with a spatial resolution as high as 60 nm^4^. The strain and orientation resolution of DFXM has been reported to be 10^−5^ and 0.001°^5–7^, respectively (compared to, e.g., electron backscatter diffraction, EBSD, angular resolutions of 0.1–0.3°^8^).

**Figure 1:**
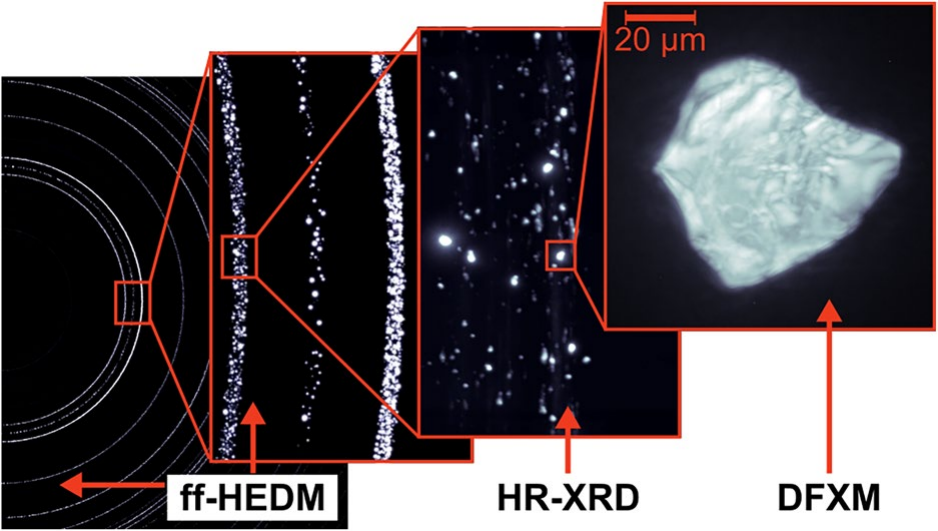
MX-ray diffraction microstructure imaging using far-field high-energy diffraction microscopy (ff-HEDM), high-resolution X-ray diffraction (HR-XRD), and dark-field X-ray microscopy (DFXM).

While high-resolution techniques like DFXM reach higher spatial resolutions, they naturally capture smaller fields of view. Thus, it is often desirable to pair DFXM with a larger field-of-view technique to elucidate, e.g., the grain network’s connectivity and/or dynamics. Larger field-of-view/lower-resolution techniques include diffraction contrast tomography (DCT), near-field or far-field high-energy diffraction microscopy (nf- or ff-HEDM), and 3D X-ray diffraction (3DXRD). In this work, we combine DFXM with a technique called high-resolution X-ray diffraction (HR-XRD). Whereas DCT, HEDM, and 3DXRD require coarse, relatively undeformed grains, HR-XRD can be used to gain insight into the dynamics of small, heavily deformed grains with temporal resolution on the order of seconds to minutes (at the cost, however, of a full grain mapping/indexing—see Section “High-resolution X-ray diffraction (HR-XRD)”). In this experiment, this combination of techniques allowed us to zoom in and out of reciprocal space, as demonstrated in Fig. 1. In the “zoomed out” mode, we measured a sampling of {101}-type Bragg reflections with HR-XRD. Then, we “zoomed in” and examined a single grain’s Bragg reflection with DFXM. In this work, this multiscale approach is used to achieve a multiscale characterization of individual grains during in-situ annealing to study static recrystallization in a Mg–3.2Zn–0.1Ca wt.% (ZX30) alloy.

Recrystallization is of modern importance to the advancement of high-strength lightweight magnesium (Mg) alloys, which have substantial potential for reducing the weight of automobiles and other transportation systems to improve fuel economy and reduce greenhouse gas emissions^9^. A major barrier, however, is the strong crystallographic texture of rolled Mg alloy sheet, which can lead to anisotropy and poor formability. Recent investigations show that annealing can be used to weaken the texture of deformed Mg alloys via recrystallization^10–17^ in alloys with common (i.e., not rare-earth) elements such as Zn and Ca^18–23^. This research shows that are at least two necessary steps for texture weakening in these alloys: a relatively weak as-deformed texture and then an annealing step resulting in static recrystallization^19,24,25^.

During recrystallization, the microstructure evolves quickly and contains both small and large grains and both heavily deformed grains and recrystallized grains. Thus, it can be challenging to characterize the microstructure evolution with a single technique. Common approaches include EBSD and transmission electron microscopy (TEM). EBSD results can provide the grain structure, grain orientation, and grain misorientation or orientation spread with spatial resolutions on the order of tens of nanometers, but EBSD has surface preparation requirements that make in-situ annealing challenging (though not impossible, e.g.^26^) because of oxidation. TEM can characterize structures on the order of Angstroms, providing details on the dislocation networks inside a grain (e.g.^27^). However, it can be challenging to produce meaningful statistics with the small fields of view accessible with TEM, and very thin samples and near-surface regions may not be representative of the bulk.

Beyond electron microscopy, X-ray-based techniques can be used to provide new insights into recrystallization. In a recent study, researchers used ff-HEDM to study the emergence and evolution of recrystallized grains in a Mg–3.2Zn–0.1Ca wt.% (ZX30) alloy during static recrystallization but could only characterize recrystallized grains when they were large enough to be detectable (~ 10 μm)^23^. Other recrystallization-specific examples include studies of the nucleation growth kinetics of recrystallized grains in 90% cold-rolled aluminum^28^, the nucleation of recrystallized grains out of the deformed matrix in 30% deformed aluminum^29^, a map of recrystallized grains in a 3D volume of partially recrystallized aluminum^30^, and simultaneous recovery and recrystallization in low-temperature annealing of an aluminum wire^31^. Despite these examples, there is still room for improvement. For example, the length scales associated with recrystallized grain nuclei are nanoscale, whereas, e.g., ff-HEDM has a spatial resolution of ~ 10 µm. Temporal resolution is also an inherent challenge to techniques that require a 360° sample rotation. Finally, the ability to apply these techniques to highly-deformed materials is difficult due to spatial resolution limitations and the challenges they pose to peak-finding and peak-fitting procedures. This is why here, we opt to combine DFXM with HR-XRD (more in Section “High-resolution X-ray diffraction (HR-XRD)”).

In this work, HR-XRD was used to measure the evolution of relative grain volume for > 8000 grains, and DFXM was used to map the internal orientation and elastic lattice strain across two different grains with 100 nm spatial resolution: one that is newly recrystallized and one that is pre-existing, i.e., exists from the as-deformed state. By combining HR-XRD and DFXM, we demonstrate the ability to take fast, large field-of-view, in-situ measurements while pausing to “zoom in” to investigate select grains of interest with higher spatial resolution, resulting in a statistical understanding of recrystallization kinetics as well as a high-resolution understanding of the internal grain structure. This combination of techniques demonstrates the ability to trade field of view and acquisition speed for spatial resolution within a single experiment.

## Methods

### Multiscale X-ray diffraction imaging overview

**Figure 2:**
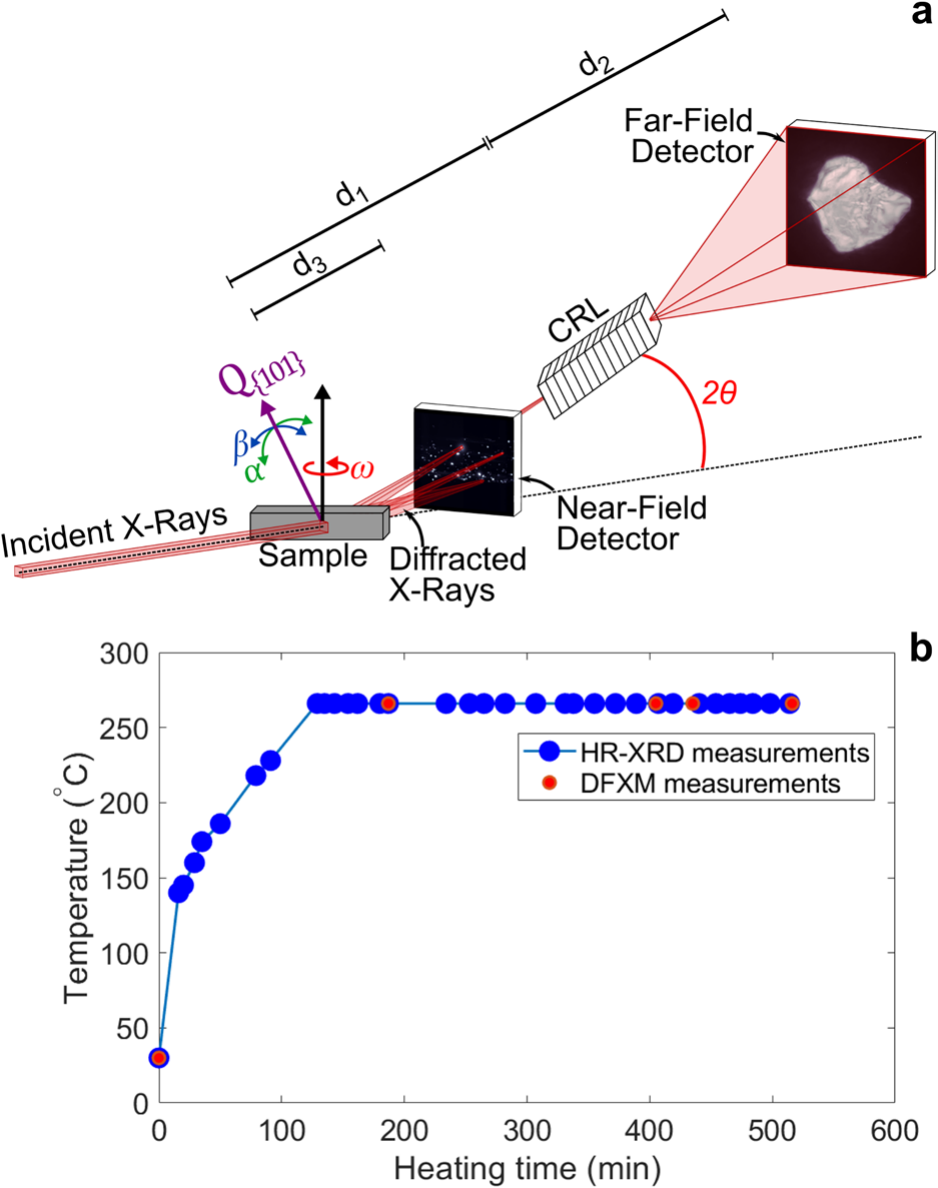
Overview of experiment. (**a**) Schematic of the high-resolution X-ray diffraction (HR-XRD) and dark-field X-ray microscopy (DFXM) setup including the incident X-ray beam, sample, diffracted X-rays, CRL (compact refractive lens) objective, and detectors. (**b**) HR-XRD and DFXM measurement times and temperatures.

The HR-XRD and the DFXM measurements were taken during a single experiment on ID06-HXM^32^ at the European Synchrotron Radiation Facility (ESRF) using an X-ray energy of 17 keV and a beam size of 200 µm × 200 µm^2^. A schematic of the experimental setup is shown in Fig. 2. Our HR-XRD setup (Fig. 2) consisted of a diffraction camera consisting of a scintillator and a charge-coupled device (CCD), which is fiber-taper couple FreLon CCD camera with an effective pixel size of 0.622 µm and a field of view of 94.6 µm × 94.6 µm. This diffraction camera is located 135–150 mm downstream of the sample (d_3_ in Fig. 2). The detector captured a field of view in reciprocal space equivalent to 3° in the azimuthal direction and 6° about the vertical axis (ω in Fig. 2) of the {101} Debye–Scherrer ring, which was located at a nominal 2θ of 17.21°. During the 6° sample rotation, images were integrated over every 0.03°, resulting in 200 images per measurement. Thus, the total acquisition time for each measurement was 100 s.

### High-resolution X-ray diffraction (HR-XRD)

3DXRD is a class of techniques wherein the sample is illuminated by monochromatic, collimated, X-rays, and diffraction patterns are continuously recorded as the sample is rotated 360°^33–35^. There are two common varieties of 3DXRD: far-field and near-field. With far-field measurements, the detector is placed in the far-field condition where the Fresnel number, $r$, is typically much less than 1^36–38^. With near-field measurements, the detector is placed in the near-field condition ($Fr>>1$)^33,39^. With high-resolution 3DXRD (HR-3DXRD), the detector is placed in the intermediate-field condition ($Fr\approx 1$)^40^. HR-3DXRD uses a high-resolution detector that is tessellated over the full circumference of multiple Debye–Scherrer rings, and the images are stitched together in post-processing to form a complete picture of reciprocal space. This procedure was recently developed by Kutsal et al.^41^. The advantage of HR-3DXRD is a gain in sensitivity to reciprocal space, making it easier to decipher small, highly deformed grains, subgrains, etc.

In this work, we use HR-XRD, which is HR-3DXRD with two modifications: First, we did not tesselate the detector. Instead, we used a high-resolution detector to zoom into a subset of Bragg reflections appearing in one particular Debye–Scherrer ring. Second, we do not rotate the sample 360°, but only ± 3°. These two modifications limited our field of view to only a subset of grains and prevented us from fitting the grains’ orientation, strain tensor, or position (hence the choice of name of HR-XRD and not HR-3DXRD), but they enable a faster data acquisition speed to allow for in-situ measurements while still providing a sufficient number of Bragg reflections for a statistical analysis. The same procedure was employed by Ahl et al. to study subgrain dynamics during recovery in a 50% recrystallized aluminum alloy in^42^.

### Dark-field X-ray microscopy (DFXM)

During the in-situ annealing HR-XRD experiment, we “zoomed in” to a particular Bragg reflection using DFXM. DFXM is a high-resolution DMI technique that can be used to observe nano/microstructure evolution with up to 60 nm spatial resolution and measure local distortions in strain and orientation on the order of 10^−5^ and 0.001°, respectively, over mesoscopic fields of view^5–7^. A schematic of the DFXM setup is shown in Fig. 2. First, the incident X-ray beam is focused using a CRL condenser consisting of 58 one-dimensional Be lenses with 100 µm apex radius of curvature. Then, the sample is adjusted such that a strong {101}-type Bragg reflection is positioned at the top of the {101} Debye–Scherrer ring. This diffracted beam is then aligned and focused by a CRL functioning as an objective lens manufactured by RXOptics and consisting of 88 two-dimensional Be lenses with a 50 µm apex radius of curvature. Once a particular Bragg reflection is aligned, it is located on a far-field detector located 5.3 m from the sample The far-field detector consisted of a 10 mm LuAG:Eu scintillator screen, an optical microscope with Olympus UIS2 UplanSApo 10×/0.40 objective, and an Olympus U-TLU-1-2 tube lens yielding an effective pixel size of 1.4 µm. Intragranular variations in crystallographic orientation are measured by tilting the sample in β and α about two perpendicular axes (see Fig. 2). Intragranular variations in elastic lattice strain in the {101} lattice plane normal direction are measured by tilting the sample α and the objective lens pitch by Δ2θ. The spatial resolution of the DFXM technique is 1.4 µm $\frac{{d}_{1}}{{d}_{2}}$, where 1.4 µm is the effective pixel size on the far-field detector, ${d}_{1}$ is the distance from sample to objective, and ${d}_{2}$ is the distance from objective to detector. In our experiment, ${d}_{1}$ = 274 mm and ${d}_{2}$ = 4,713 mm, yielding a magnification of 17.2 and a spatial resolution of 81 nm per pixel. However, due to lens defects, the true spatial resolution is approximately 100 nm.

### Material preparation

**Figure 3:**
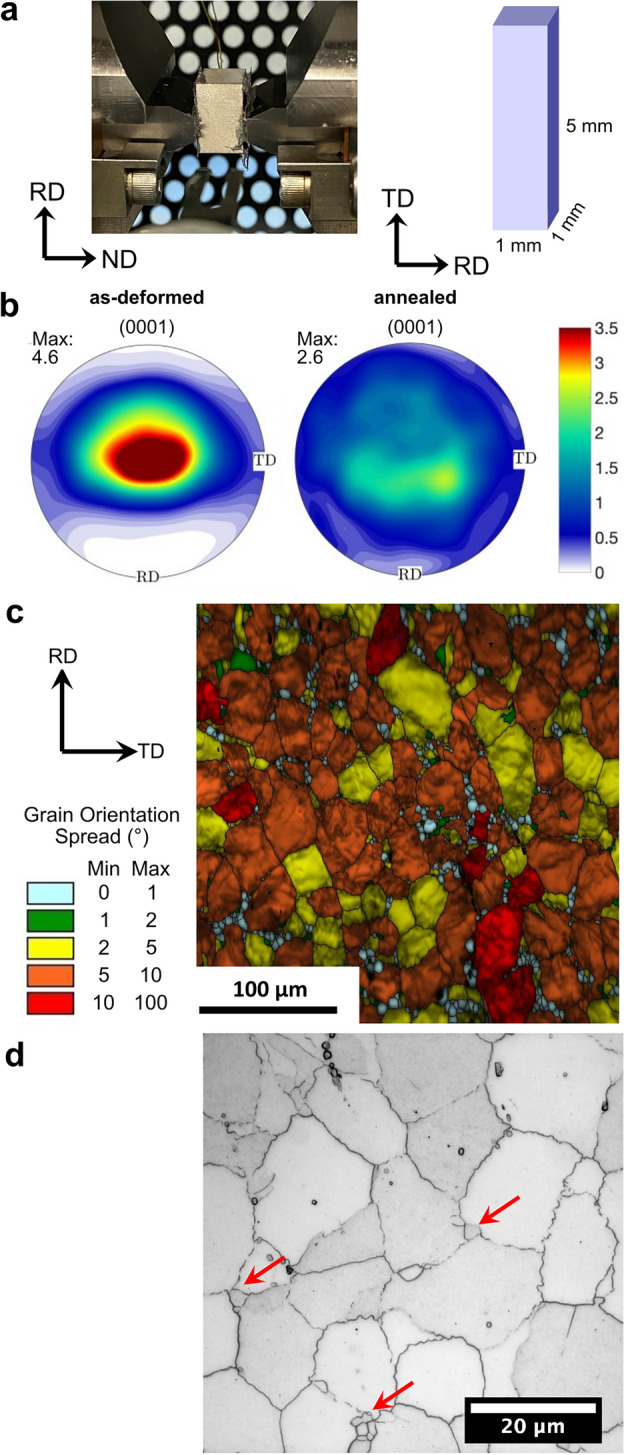
Overview of the sample. (**a**) Sample during deformation inside the Gleeble thermomechanical simulator and schematic of the sample geometry. (**b**) Basal pole figures showing the texture weakening that occurs in the ZX30 alloy during annealing. The color bar refers to multiples of random distribution. (**c**) Representative grain orientation spread (GOS) of the as-deformed microstructure. (**d**) Representative optical microscopy images of the as-deformed microstructure.

A hot-compressed Mg–3.2Zn–0.1 Ca wt.% (ZX30) alloy sample was prepared using a Gleeble thermomechanical simulator to replicate hot rolling via hot plane strain compression. The ZX30 alloy was selected, because past work has shown that this alloy demonstrates the desirable texture weakening phenomenon as a result of static recrystallization that is the motivation for this study. Ten passes of plane strain compression were applied at 350 °C with 0.2 strain per pass and a final strain value of 2.2 (80%). Before the first pass, the sample was heated to 350 °C for 10 min, and the temperature was maintained at 350 °C throughout the operation, including between passes. After the tenth (final) pass, the sample was air quenched within 30 s to room temperature to limit recrystallization in the final pass. The deformed sample was machined into 1 × 1 × 5 mm^3^ rectangular bars using electron discharge machining, as illustrated in Fig. 3a for the experiment presented in this work. The transverse direction (TD) is parallel to the 5-mm axis, and the normal direction (ND) is parallel to one of the 1-mm axes. The TD was perpendicular to the incident X-ray beam and parallel to the sample rotation axis during the synchrotron experiment.

The texture of a sample with the same composition and thermomechanical history was measured in the as-deformed and annealed conditions (120 min at 300 °C) using EBSD. The results are shown in Fig. 3b. The results demonstrate a moderate basal texture in the as-deformed condition and a weaker “rare-earth” or “quad” texture^22^ after annealing, representing the texture weakening phenomenon that motivates this work.

Figure 3c,d shows grain orientation spread (GOS) and optical measurements of the sample in the as-deformed condition. The results show the presence of a small fraction of small low-GOS grains along grain boundaries and at triple points (red arrows in the optical map and shown in blue in the GOS map), which could be recrystallized grains that formed during the Gleeble processing. Thus, there may have been a small fraction (3–6%) of dynamic recrystallization that occurred during deformation. A study on Mg–3.2Zn–0.1Ca (ZX30) alloys after hot plane strain compression using the Gleeble thermomechanical simulator also showed evidence of dynamic recrystallization^22^.

### Sample heating

A hot air blower positioned 5 mm from the sample was used to perform the heating. The air blower was powered by a fast-programming Delta Electronika SM15-400/P-166 and regulated by a Eurotherm 2408 unit. The blower was used to anneal the sample for a total of 514 min, during which time we collected 33 HR-XRD measurements. The sample was continuously heated at a rate of 10 °C/min for the first 129 min. Once the sample temperature reached 266 °C at 129 min, the sample temperature was maintained at 266 °C degrees for an additional 385 min (with a total heating time of 514 min). Prior to conducting each DFXM measurement, the sample was cooled to room temperature. Upon completion of each DFXM measurement, the sample’s temperature was back to 266 °C at a rate of 150 °C/min, to continue with the annealing process.

To calibrate the sample temperature, we used the thermal expansion of the {101} Debye–Scherrer ring assuming a thermal expansion coefficient of 25 × 10^−6^ K^−1^^43^. Note: the illuminated volume could have changed slightly due to thermal expansion of the sample. Using this thermal expansion coefficient, we estimate that ~ 93% of the illuminated volume remained in view for the entire experiment. Figure 2b shows the sample heating profile during the synchrotron experiment and where the HR-XRD and DFXM measurements were collected.

### HR-XRD data analysis

From the HR-XRD images, the shape, integrated intensity, and position of each Bragg reflection within the observed field of view in reciprocal space was measured at each annealing time step. To do so, the images underwent the following processing steps: (1) Uniform shifts in the Bragg reflection positions due to thermal expansion or sample motion were corrected. (2) Image noise was removed using a median background subtraction with a running median of 20 images. (3) Any remaining image noise, as well as the diffuse (powder-type) scattering caused by highly deformed, nanocrystalline domains/grains below our spatial resolution was removed using a bilateral filter. (4) Blob detection, a Python OpenCV library, was used to measure the outline and position of each Bragg reflection occurring on each image. (5) Any Bragg reflections occurring at the same detector position across multiple images corresponding to different ω positions were assigned as a single Bragg reflection. (6) For each Bragg reflection, the centroid (in *x*, *y*, ω), area *A*, and integrated intensity *I* were measured. As demonstrated in^42^, the relative grain volume $V$ and grain radius $R$ can be related to the integrated intensity, $V\propto I$, with $R\propto \sqrt[3]{I}$. (7) Finally, to track a Bragg reflection from one time step to the next, the following procedure was used. If two Bragg reflections in consecutive measurements had centroid positions that differed by ≤ 0.06° (twice the ω increment) and ≤ 15 pixels in the *x*,*y* detector plane, then they were accepted as the same Bragg reflection at different time steps. Because we are only observing a small window of reciprocal space, we assumed that each Bragg reflection corresponds to a specific grain. (Bragg reflections very close to or cut off by the image edges were not included.) Potential sources of error include inaccuracies in these image processing and segmentation procedures, especially due to very weak, diffuse Bragg reflections caused by severe deformation. Other potential sources of error include classification errors, e.g., when a Bragg reflection, originally positioned near an edge of the detector, moves off the detector.

The percent growth rate was calculated as $\frac{{V}_{t+1}-{V}_{t}}{{V}_{t}\Delta t}\times 100\%$, where ${V}_{t+1}$ is the relative grain volume at time step $t+1$, ${V}_{t}$ is the relative grain volume at time step $t$, and $\Delta t$ is the annealing time between these two time steps.

### DFXM data analysis

The elastic lattice strain and relative orientation are measured from the sample tilting angles β and α and the objective lens pitch 2θ. For both scan types, a 2D intensity distribution is recorded at each pixel, and the center of mass of this distribution is calculated. Elastic strain ${\varepsilon }_{hkl}$, is calculated from the nominal d-spacing, ${d}_{hkl}^{0}$, and the strained d-spacing, ${d}_{hkl}$, X-ray wavelength, $\lambda$, the nominal 2θ, and the change in pitch of the objective, Δ2θ: ${d}_{hkl}=\frac{\lambda }{2 sin\frac{\Delta 2\uptheta +2\uptheta }{2}}$, ${d}_{hkl}^{0}=\frac{\lambda }{2 sin\frac{2\uptheta }{2}}$, ${\varepsilon }_{hkl}=\frac{{d}_{hkl}-{d}_{hkl}^{0}}{{d}_{hkl}^{0}}$. For more detail regarding the analysis procedures, see, e.g.^7,32^. For the pre-existing grain, the orientation scan consisted of a 2D mesh composed of diffraction images taken at α tilt angles of ± 0.05° in 21 steps meshed with β tilt angles of ± 0.025° in 11 steps. For the recrystallized grain, the orientation scan consisted of a 2D mesh composed of diffraction images taken at α tilt angles of ± 0.09° in 45 steps meshed with β tilt angles of ± 0.2° in 11 steps. For the pre-existing grain, the elastic lattice strain scan consisted of a 2D mesh composed of diffraction images taken α tilt angles of ± 0.03° in 21 steps meshed with Δ2θ tilt angles of ± 0.05° in 11 steps. For the recrystallized grain, the elastic lattice strain scan consisted of a 2D mesh composed of diffraction images taken α tilt angles of ± 0.06° in 21 steps meshed with Δ2θ tilt angles of ± 0.05° in 11 steps.

## Results and discussion

**Figure 4:**
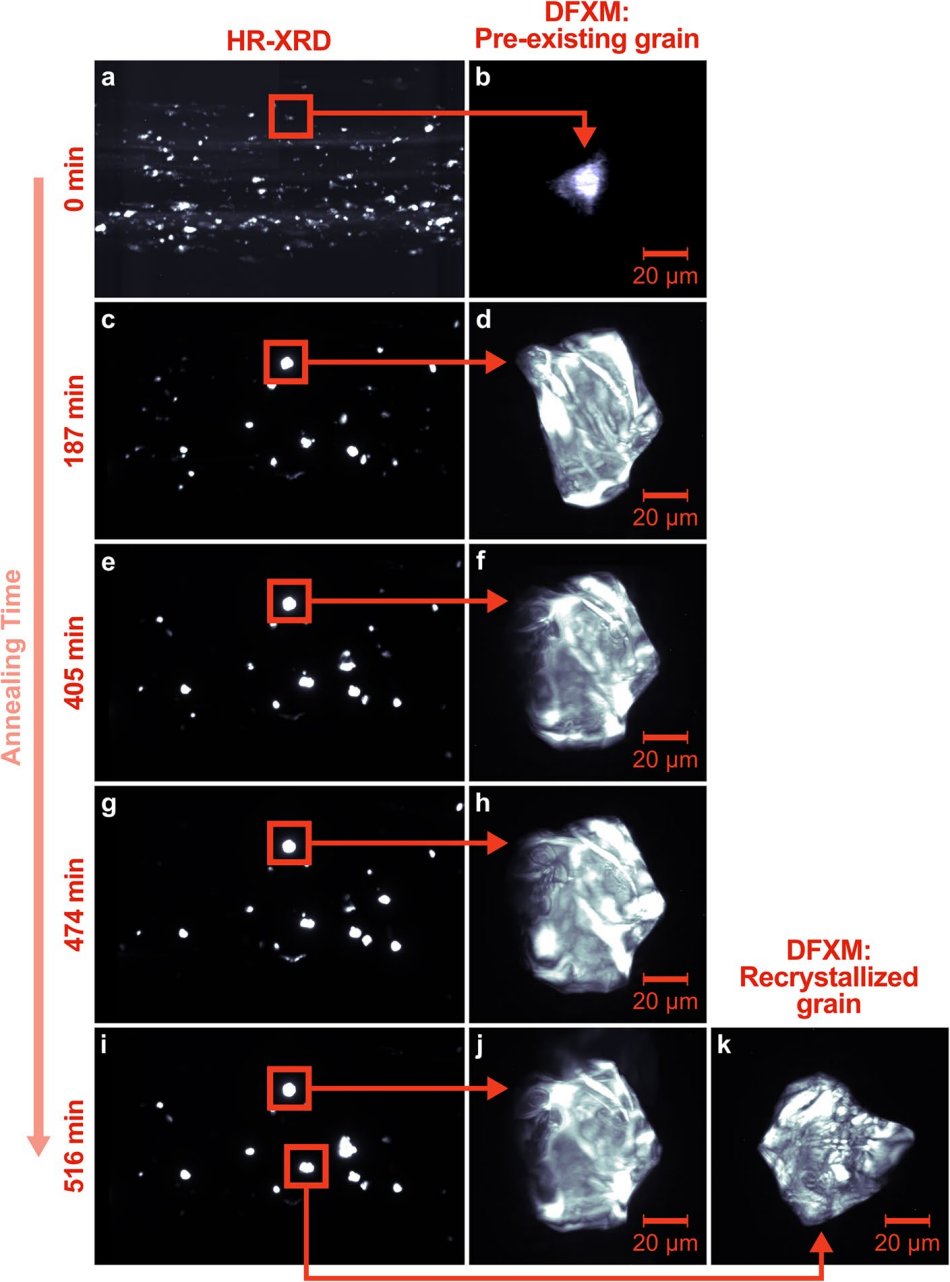
Demonstration of the “zoom in/zoom out” capabilities of HR-XRD combined with DFXM. HR-XRD images (**a**,**c**,**e**,**g**,**i**) and DFXM images (**b**,**d**,**f**,**h**,**j**,**k**) are summed over all tilt angles at 0, 187, 405, 474, and 516 min annealing. DFXM measurements were taken on one pre-existing grain throughout annealing (**b**,**d**,**f**,**h**,**j**) and one recrystallized grain at the end of annealing (**k**).

**Figure 5:**
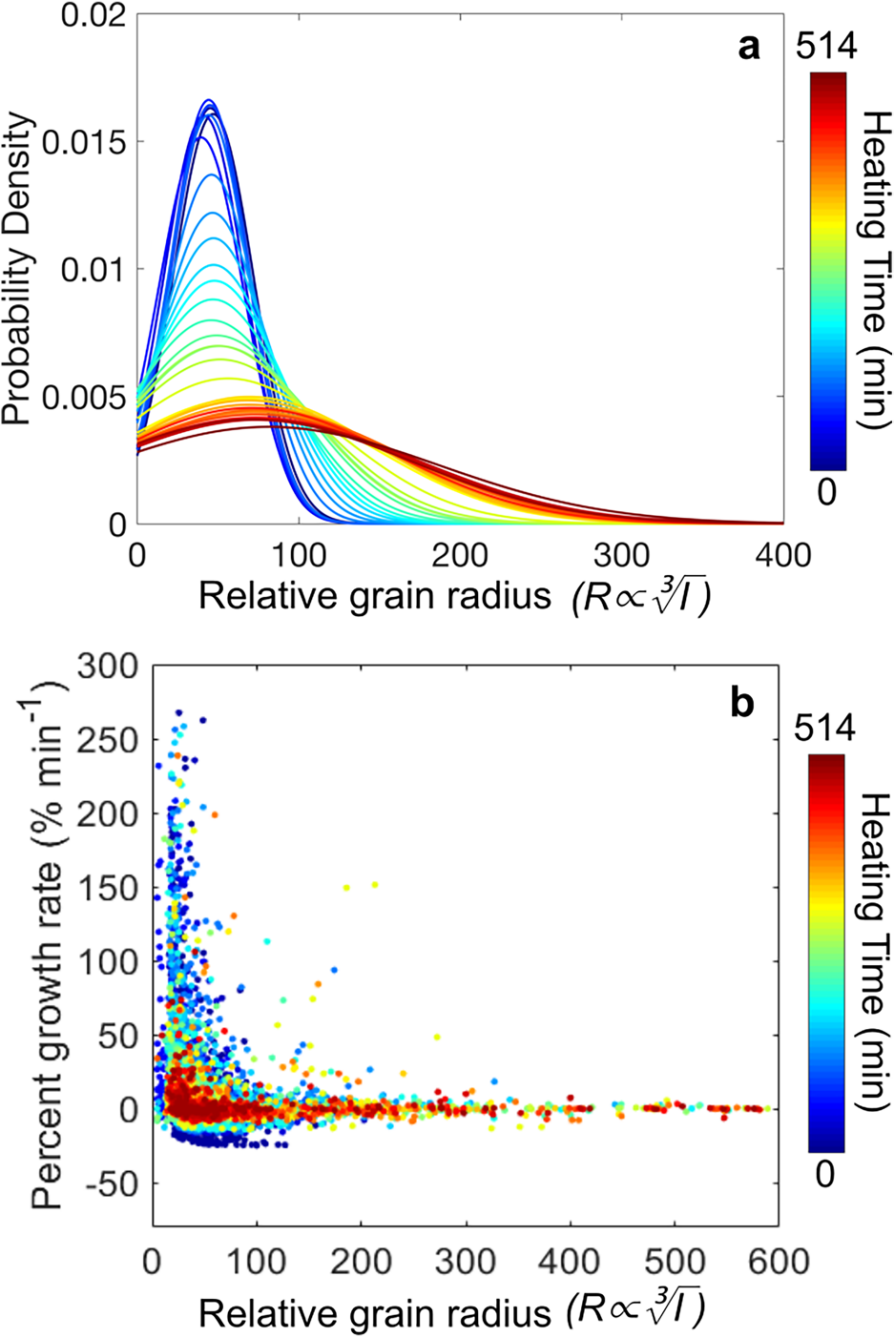
Size evolution of all grains during annealing. (**a**) Probability density of relative grain radius (measured as proportional to the cube root of relative intensity of the Bragg reflection). (**b**) Percent growth rate for all grains throughout annealing.

Select HR-XRD and DFXM images are shown in Fig. 4 demonstrating the “zoom in/zoom out” capabilities of combining these two techniques. Figure 4 shows total intensity images, i.e., the intensity is summed over the rotating or tilting that occurred during the scan. The complete HR-XRD time series is provided as a video in the Supplemental Material. In the first measurement 1326 grains were detected. As annealing proceeds, the number of grains can be seen decreasing, and select grains become very large, as can be observed by the high relative intensity of their Bragg reflections. At the end of annealing, 153 grains exist. The relative grain radius of each Bragg reflection measured with HR-XRD is shown in Fig. 5a. The relative grain radius (measured as proportional to the cube root of integrated intensity of the Bragg reflection) can be seen spreading and shifting toward larger volumes with annealing time. The Supplemental Material also contains videos showing the evolution of the relative grain radius and ω position across all HR-XRD measurements. (Note: As previously mentioned in Section “HR-XRD data analysis”, the relative grain radius is calculated by the cube root of the normalized, integrated intensity of the Bragg reflection. Thus, the value is nondimensional and there is no unit assigned) (Supplementary Video 1, 2).

The percent growth rate versus relative grain radius is shown in Fig. 5b. The marker color corresponds to annealing time. In the as-deformed state (dark blue), the initial microstructure consisted of only relatively small grains, many of which show rapid growth with a large spread in percent growth rate. As the annealing process progresses and the grains grow, there are more large grains, and the growth rate decreases. The same result was observed experimentally for the ZX30 alloy in Roumina et al.^23^. One interesting observation is that recrystallization is still active even after several hours of heating. In the final time step, a total of 15.7% of the grains were newly recrystallized (i.e., did not exist in the previous annealing step). In the final annealing step, the annealing temperature is higher than in the as-deformed step, but it shows a relatively lower percentage growth rate than the microstructure in the early annealing (See in Fig. 5b). Additionally, the microstructure at the end of annealing (dark red) has both large grains and small grains.

By tracking each grain from its first emergence to the final annealing time step, we can identify each grain as a pre-existing grain, a recrystallized grain, or a consumed grain at any given time step. The definitions for each of these terms are given below.

- *Pre-existing grains*: Grains that have been detected from the first time step and continue to be detected
- *Recrystallized grain*s: Grains that were not detected in the first time step but emerged at some point during the annealing process
- *Consumed grain*s: Grains that were detected at a previous time step but are no longer detected

**Figure 6:**
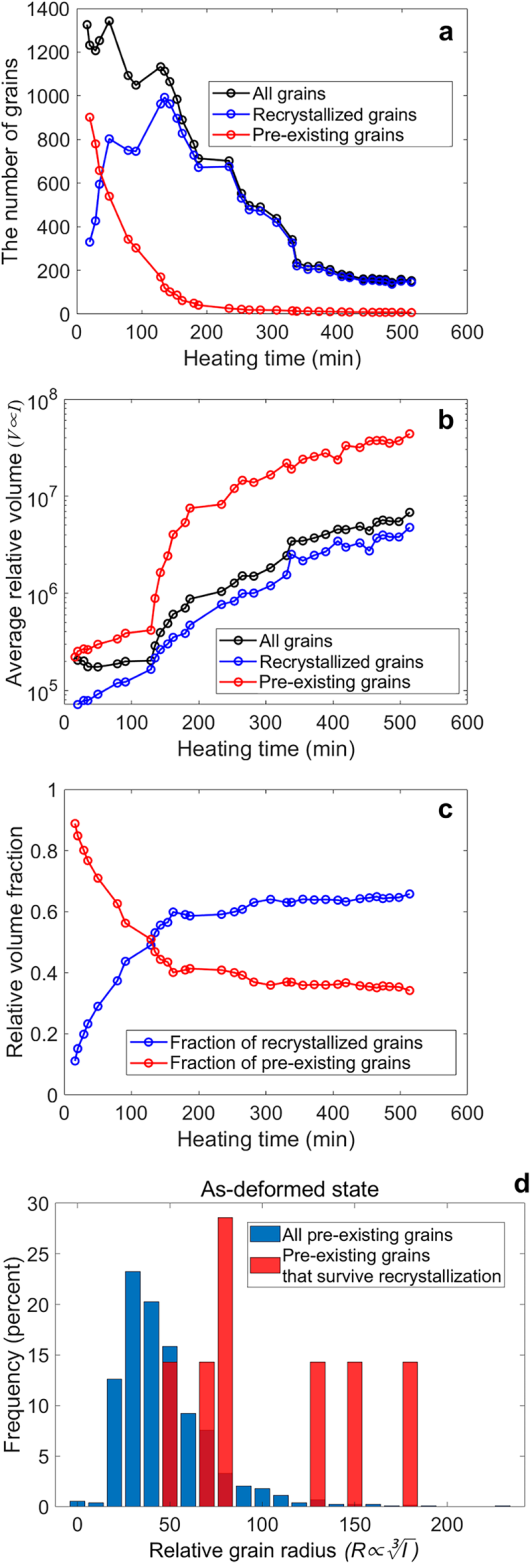
The amount of recrystallized versus pre-existing grains during annealing. (**a**) The number of grains, (**b**) the average relative volume, and (**c**) the relative volume fraction of all grains (black), recrystallized grains (blue), and pre-existing grains (red) versus heating time. (**d**) Frequency plot showing the size distribution of in the very first HR-XRD measurement, where all of the grains detected are included in the blue distribution and only the grains that eventually “survive” recrystallization are included in the red distribution.

The results are presented in Fig. 6. Figure 6a shows the total number of grains (black), the number of recrystallized grains (blue), and the number of pre-existing grains (red) measured at each time step. In the initial (as-deformed) state, 1326 grains were measured. Upon heating, the number of pre-existing grains (red) decreases as the number of recrystallized grains (blue) increases. This is the process of recrystallization where the original, deformed volume is consumed by recrystallized, relatively undeformed grains, lowering the overall strain energy of the system. At 135 min of annealing, the number of recrystallized grains is at its peak, after which it begins to decrease as some grains are consumed and other grains grow. At the final time step, 153 grains were measured. Of these 153 grains, only 7 are pre-existing grains, i.e., grains that existed from the as-deformed condition and “survived” the annealing process to the end. The other 146 grains are all recrystallized grains that did not exist in the as-deformed condition and emerged during annealing.

Figure 6b shows the average relative volume of all grains (black), recrystallized grains (blue), and pre-existing grains (red) measured at each time step. The average relative volume of all categories of grains increases with annealing time. A steep increase in average grain volume can be observed around 129 min. This time step corresponds to the point where the sample reached its target temperature of 266 °C. Notice that the pre-existing grains (red) consistently have larger relative volumes than the recrystallized grains (blue). Figure 6c shows the relative volume fraction of recrystallized (blue) versus pre-existing (red) grains. Notice that the pre-existing grains account for a significant fraction of the total volume at all time steps. Even at the end of annealing, when only 7 out of 153 total grains are “pre-existing grains”, these 7 pre-existing grains account for 34% of the total volume. Because pre-existing grains will carry with them the strong crystallographic texture of the as-deformed condition, this observation suggests that the “survival” of pre-existing grains during the recrystallization process may have a significant effect on the final recrystallization texture of the sample^44,45^. This is discussed more below.

Because the results shown in Fig. 6a–c suggest that even a small number of pre-existing grains can have a significant effect on the final recrystallization texture of the sample, it is useful to ask the question: Are there any indicators we can use to predict which grains in the as-deformed condition will survive recrystallization? Fig. 6d shows the distribution of relative grain radius of all 1,326 grains (blue) in the as-deformed state. (Recall: The grain radii are calculated from the relative grain volumes, which are taken to be proportional to the relative/normalized intensity of the grain’s Bragg reflections and are thus dimensionless.) Also shown is the distribution of relative grain radius for only the 7 pre-existing grains that survive the recrystallization process (red). The results show that the 7 pre-existing grains that survive recrystallization are larger than most of the grains in the as-deformed condition. The average relative grain radius of these 7 grains is 105, whereas the average relative grain radius of all grains in the as-deformed state is 47. Even the smallest of these 7 grains has a larger radius than the average radius of all grains in the as-deformed state. (Note: In order to provide both histograms on the same plot for direct comparison, the histogram bars are partially transparent. Thus, the dark red is caused by overlapping blue and red bars.)

These observations suggest that the initial grain size may play an important role in determining which grains will survive recrystallization, with larger grains more likely to survive than smaller grains. However, it is unclear whether or not these initially large grains had recrystallized and grown during the hot deformation process. As shown in Fig. 3c,d, the as-deformed microstructure shows the presence of a small fraction of small low-GOS grains along grain boundaries and at triple points (red arrows in Fig. 3d and shown in blue in Fig. 3c), which could be dynamically recrystallized grains that formed during the Gleeble processing. A study on ZX30 alloys after hot plane strain compression using a Gleeble thermomechanical simulator also showed evidence of dynamic recrystallization^22^. These results suggest that the 7 pre-existing grains could be grains that recrystallized early (i.e., dynamically recrystallized during the deformation process) and grew early.

In the work of Liu et al.^46^, researchers used in-situ nf-HEDM to show that grains that recrystallized early tended to dominate the final microstructure, and grains that appeared later tended to be consumed (presumably due to their relatively small volumes). These observations align with the present work, where the 7 initially large “pre-existing” grains not only survive, but eventually account for 34% of the final volume. These findings may suggest that grains that recrystallize and grow early may have a significant influence on shaping the final microstructure. As remarked in^46^, this insight opens the possibility of controlling the density of these early-recrystallized grains to adjust the final microstructure.

**Figure 7:**
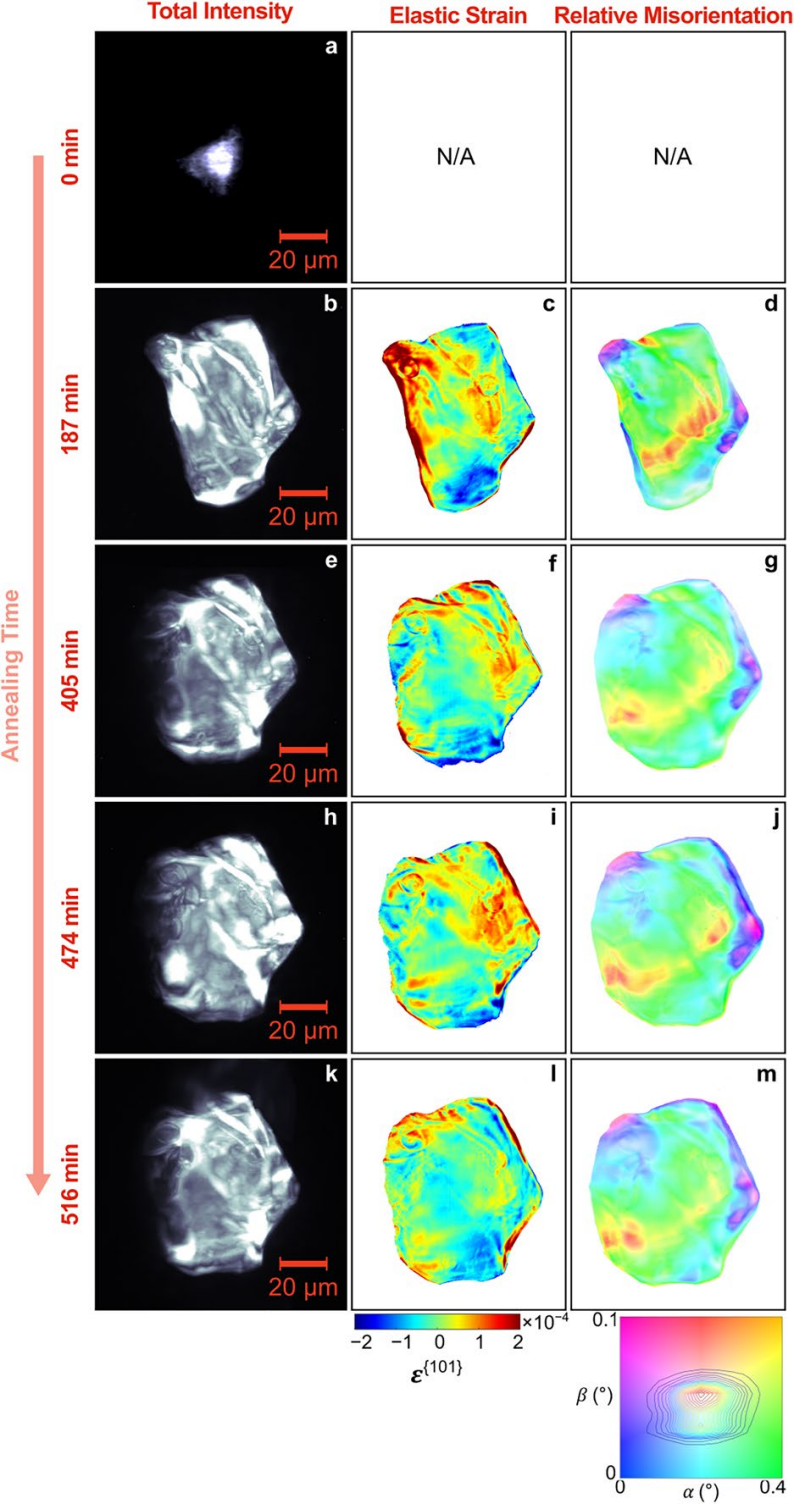
Spatially resolved maps of the total intensity (first column), elastic strain ${\upvarepsilon }^{\{101\}}$ (second column), and relative misorientation (third column) of one of the “surviving” pre-existing grains.

**Figure 8:**
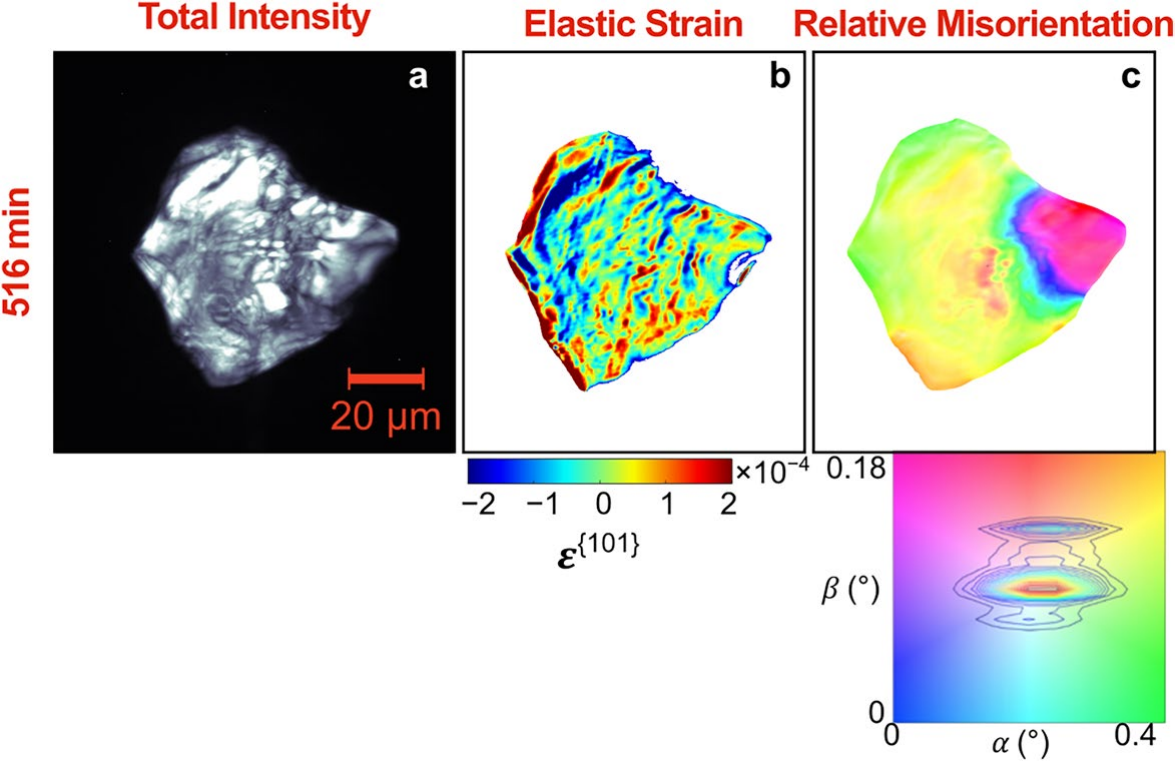
Spatially resolved maps of the total intensity (first column), elastic strain ${\upvarepsilon }^{\{101\}}$ (second column), and relative misorientation (third column) of a recrystallized grain at 516 min annealing.

As previously mentioned, during the in-situ HR-XRD experiment, we paused the annealing process (i.e., cooled the sample to room temperature) and “zoomed in” to specific grains using DFXM (Fig. 4). DFXM measurements were taken on one grain in the as-deformed condition, and DFXM measurements were taken on this same grain at 187, 405, 474, and 516 min annealing: This was a pre-existing grain that survived the annealing process. Finally, at 516 min annealing time, DFXM measurements were also taken on a recrystallized grain. The DFXM results are shown in Figs. 7 and 8.

Figure 7 shows spatially resolved maps of the relative intragranular misorientation for the pre-existing grain at different annealing times. (Note: The as-deformed condition is omitted, as the grain was too small and deformed to produce a meaningful reconstruction.) Notice that the pre-existing grain had grown to almost its final size by 187 min, and that its left boundary grows outward between 187 (Fig. 7b) and 405 min (Fig. 7e). This growth coincides with visible changes in the internal elastic strain and relative misorientation. Specifically, the left grain boundary has a high local strain field prior to this growth, and after the growth event the strain field is gone (i.e., the material has relaxed). Furthermore, the grain boundary is observed to grow in the direction of its center of curvature, which is more consistent with classical understanding. After 405 min, the size, shape, internal elastic lattice strain, and internal relative misorientation does not change significantly, though there appears to be some relaxation between 474 min (Fig. 7h) and 516 min (Fig. 7k), perhaps coming from a growth event out of the plane of these measurements. Because we know this to be one of only seven pre-existing grains that survived annealing, the stability of the growth and internal structure of this grain may be important to understanding which pre-existing grains will survive (and how they survive) annealing. Based on these results, we conjecture that the pre-existing grains have more driving force for grain boundary migration compared to its surroundings versus grains that do not survive annealing (since it survives), that this growth starts early in the recrystallization process, and that these pre-existing grains may thus recover and grow simultaneously (i.e., there is a collective motion of dislocations that annihilate themselves within the grain, but also moves the boundary forward as it grows).

Figure 8 shows the spatially resolved maps of relative misorientation across the recrystallized grain at the end of annealing. This particular recrystallized grain was observed to have a subgrain. Even though this subgrain is only roughly 0.1° misaligned from the parent grain, it was observable even in the HR-XRD measurements. Subgrains can serve as recrystallization nucleation sites (or the nucleation seeds themselves) as shown in many works, including the DFXM work of Yildirim et al.^47^. However, it is interesting to see that the two subgrains combine to form such an equiaxed grain shape. It should be noticed that, even though recrystallized grains are generally assumed to have zero internal elastic lattice strain and misorientation, the high angular resolution capability of DFXM shows that even recrystallized grains can have significant internal lattice distortion. Also notice that the recrystallized grain and the pre-existing grain have roughly the same internal strain spread. While it is acknowledged that no grain is perfectly homogeneous, the unique angular sensitivity of DFXM allows for the characterization of the local strain and misorientation map, even within recrystallized grains, revealing the presence of inhomogeneities, such as local elastic strains, rotations, and low-angle grain boundaries.

The results demonstrate the advantage of DFXM’s high angular resolution (capable of detecting strain variations as small as 10^−5^^32^) for studying grains with low internal distortion such as recovered and recrystallized grains, making it a valuable tool for understanding recrystallization, recovery, and grain growth. In both cases shown above, DFXM is able to show that even recrystallized grains can have heterogeneous internal stresses and orientation distributions. Recent research has also shown that significant local stresses exist within individual recrystallizing grains even after high-temperature heat treatments^48^.

## Conclusion

A combination of HR-XRD and DFXM was used to study recovery and recrystallization in a hot-compressed Mg–3.2Zn–0.1Ca (ZX30) alloy during in-situ annealing. HR-XRD was used to measure the evolution of relative grain volume for over 8,000 grains, and DFXM was used to visualize and measure the spatially resolved maps of the relative misorientation and internal elastic lattice strain across the individual pre-existing and recrystallized grain at different annealing times with 100 nm spatial resolution. By combining HR-XRD and DFXM, we demonstrate the ability to take fast, large field-of-view, in-situ measurements while pausing to “zoom in” and investigate select grains of interest with high spatial resolution, resulting in a statistical understanding of recrystallization kinetics as well as a high-resolution sampling of internal recrystallized grain structure. This combination of techniques offers a way to trade field of view and acquisition speed for spatial resolution within a single experiment.

This study demonstrates the potential of multiscale DMI experiments to provide new insights into the behavior of dynamic, multiscale phenomena. Combining high-resolution/small field-of-view techniques like DFXM and BCDI with lower-resolution/larger field-of-view techniques like HR-XRD, DCT, and HEDM can provide richer insights by giving the high-resolution measurements valuable context in terms of statistical significance and the surrounding microstructure. Finally, while grain-mapping techniques like DCT and HEDM are preferable when the microstructure is resolvable, HR-XRD is a useful alternative for studying the dynamics of small and/or highly deformed grains with temporal resolution on the order of seconds to minutes.

### Supplementary Information

Supplementary Video 1.

Supplementary Video 2.

## Data Availability

The experimental datasets analyzed during the current study are available in the Materials Commons via the https://doi.org/10.13011/m3-xbjb-va37.
